# Health Tract, No. 271

**Published:** 1867-01

**Authors:** 


HEALTH TRACT, No. 271.

### INTEMPERATE WOMEN.

"GIVE me some brandy," said she, as she seemed to be slowly recovering from a swoon in a bookstore. She conversed fluently, was highly educated and wrote a beautiful hand. Her husband was a merchant, worth nearly half a million of dollars, and connected with some of the best families of New York. Her love of liqour was so great that every member of the household was trained to keep such a watch that it was next to impossible to obtain it under her own roof. Friends and relatives knew her failing so well that they habitually acted in concert with the unfortunate husband, to save his name, and their own. But now and then the fiend of drink would come upon her with such a frenzy that all the powers of her gifted mind were at such times, bent upon obtaining the means of ministering to the insatiable appetite for brandy; and one of her plans was to step into a store where she was unknown, enter into conversation with all the grace and culture of a refined woman, and in the midst of it to feign a swoon and a slow recovery; and then, to call for brandy, as stated at first, with the perfect certainty, under the circumstances of the case, of having her wishes gratified. At times she would go to some village near New York, go from store to store, and in a short time would be carried from the street in a state of beastly intoxication. Rumor has it that a number of ladies, the daughters and wives of men of position in trade and finance and family in New York, have made application for admission into the institution at Binghamton, New York, the object of which is to make a scientific attempt to cure those who are the victims of intemperance and are willing to make an effort for their own reclamation. It is known that the wife of one of the most honored men in the nation, lately deceased, was a habitual and unreclaimable drunkard, and died such.

The early use of tea and coffee by our daughters is the first step in this direction. It is surprising how often at public and private tables when young ladies are asked how they will take their tea, "strong," is replied. Then again it is the habit of New Yorkers to have tea and coffee at luncheon; thus it is served three times a day, for it is never absent from the 5 o'clock dinner table. Another cause is that in any attack of indigestion, or the over fulness of a hearty meal, or other derangement of the stomach or bowels, brandy has become the panacea, and mothers and fathers have it at their tongues' end for all such occasions, but more especially the mothers, for they are always at home. Then again, beautiful women, women of known conversational powers, who sing well, or dance divinely, or have the reputation of being "good company," find themselves at times unfitted for the occasion, and would willingly remain at home; but from the 'must' of propriety or courtesy there is no appeal and something is taken to aid them in being 'up to the occasion.' It is on the same principle precisely that so many politicians and public speakers, and wits and poets are led into habits of intoxication. The woman of any age who finds herself drinking cold tea or coffee between meals, or of taking a glass of wine or other stimulant before 'going out,' is not far from a drunkards's grave. Nor is the politician or orator who takes a glass of brandy and water before speaking; nor the minister who before he goes into the sacred desk, feels the need of a cup of tea or coffee, or a glass of wine or a brandy toddy. The wise will be warned. He who says, "There is no danger for me," is already lost! — *Watchman and Reflector.*

